# Interspecies Differences in Cytomegalovirus Inhibition by Cardiac Glycosides—A Unique Role of the Alpha3 Isoform of the Na^+^/K^+^-ATPase Pump

**DOI:** 10.3390/v17030398

**Published:** 2025-03-11

**Authors:** Hong Mei, Hongyi Cai, Fengjie Liu, Rajkumar Venkatadri, Halli E. Miller, Angela J. Mathison, Hua-Yu Leo Wang, Simone C. Silva, George A. O’Doherty, Ravit Arav-Boger

**Affiliations:** 1 Department of Pediatrics, Division of Infectious Disease, Medical College of Wisconsin, Milwaukee, WI 53226, USA; 2 Department of Pediatrics, Division of Infectious Disease, Johns Hopkins University School of Medicine, Baltimore, MD 21287, USA; 3 Department of Surgery, Division of Research and Linda T. and John A. Mellowes Center for Genomic Sciences and Precision Medicine, Medical College of Wisconsin, Milwaukee, WI 53226, USA; 4 Department of Chemistry, Northeastern University, Boston, MA 02115, USA

**Keywords:** cytomegalovirus, cardiac glycosides, Na-K-ATPase, α3 isoform, promyelocytic leukemia protein

## Abstract

Cardiac glycosides (CGs), historically used to treat heart failure and arrhythmias, bind to the α subunit of the Na^+^/K^+^-ATPase pump and inhibit its activity. Their anticancer and antiviral activities are of interest. The α subunit of the Na^+^/K^+^-ATPase pump has four isoforms (α1–4), each with unique tissue distribution and expression pattern; their contributions to antiviral activities have not been studied. We previously reported that CGs inhibit human CMV (HCMV) in vitro but not mouse CMV (MCMV). In addition to the low affinity of mouse α1 for CGs, we hypothesized that other isoforms contribute to the anti-CMV activities of CGs. We show here that infection with HCMV significantly induced α3 in human foreskin fibroblasts, while MCMV did not induce mouse α3. Infection with guinea pig CMV (GPCMV) in GP fibroblasts also induced α3, and CGs inhibited GPCMV replication. HCMV inhibition with digitoxin reduced α3 expression. The concentration-dependent inhibition of HCMV with digitoxin analogs also correlated with α3 expression. Intriguingly, α3 was localized to the nucleus, and changes in its expression during infection and digitoxin treatment were mostly limited to the nucleus. At 4 h post-infection, α3 colocalized with immediate early 1 (IE1) and the promyelocytic leukemia protein (PML). An interaction of α3-PML-IE1 at 24 h post-infection was disrupted by digitoxin. The mRNA levels of IE1, major immediate early promoter (MIEP)-derived IE, and antiviral cytokines were reduced in infected digitoxin-treated cells. Summarized, these findings suggest a new role for α3 in the anti-HCMV activities of CGs via nuclear antiviral signaling pathways.

## 1. Introduction

Cardiac glycosides (CGs) have been used for centuries to treat congestive heart failure and arrhythmias, conditions in which they bind to the Na^+^/K^+^-ATPase pump and inhibit its activity [1,2]. The number of CGs identified in animals and plants is growing, and novel activities are becoming evident, including anticancer and antiviral [3,4,5,6,7,8]. The anticancer effects of CGs have been reported in multiple studies, and the antiviral mechanisms have gained interest during the past several years [5,9,10,11,12,13].

CGs were reported to inhibit human cytomegalovirus (HCMV) replication at nM concentrations [14,15]. The 50% effective concentration (EC_50_) of digitoxin is 23.3 ± 0.007 nM, and the CC_50_ is 2.8 ± 0.7 µM, resulting in a selectivity index of 120 [16]. Work has shown that digitoxin may have anticancer effects at concentrations commonly found in cardiac patients (0.02 to 0.033 μM) [17]. Based on the reported in vitro concentrations of HCMV inhibition by digitoxin, it may have some inhibitory effects on HCMV in vivo. Still, additional CGs with potential clinical applications are needed. In previous work, we found that digoxin, ouabain, and digitoxin inhibit HCMV replication at an early stage of infection [18], after HCMV binding and before DNA replication. Levels of immediate early (IE), early, and late viral proteins were significantly reduced in HCMV-infected cells treated with CGs. We subsequently reported that both the type of sugar and the length of the oligosaccharide chain linked to digitoxin correlate with HCMV inhibition [16].

CGs bind to the α subunit of the Na^+^/K^+^-ATPase pump, an integral membrane protein composed of two essential subunits, α and β. There are four isoforms of the catalytic α-subunit (α1, α2, α3, and α4); each isoform displays a unique tissue distribution and expression pattern, suggesting a tissue-specific role [19,20]. The α1 isoform is found in nearly every tissue, α2 is predominantly expressed in adipocytes, skeletal muscle, heart, and brain, α3 is abundant in nerve tissues and is also overexpressed in a variety of malignant human cells, and α4 is expressed exclusively in testis [3]. Mutations that result in haploinsufficiency of α3 have been linked with neurological disorders such as rapid-onset dystonia–parkinsonism [21], alternate hemiplegia of childhood [22] cerebral ataxia, early-life epilepsy, episodic apnea, and postnatal microcephaly [23].

The isoforms of the α-subunit contain the highly conserved CGs binding site, and, in most species, all four α isoforms are sensitive to inhibition by CGs. However, α1 in mice and rats has a low affinity for CGs and is resistant to inhibition by these compounds [24]. The sensitivity of tumor cells to CGs is species-dependent and has been attributed, at least in part, to the relative expression of α1/α3 isoforms [25]. The lack of α3 relative to α1 isoform in rodent tumor cells was a proposed explanation for their unresponsiveness to CGs.

We reported that, in contrast to their potent HCMV inhibition, CGs do not inhibit mouse CMV (MCMV) replication in mouse embryonic fibroblasts (MEFs) [18]. In addition to the low affinity of mouse α1 for CGs, we hypothesized that infection with HCMV induces relative expression of specific α isoforms in infected human foreskin fibroblasts (HFFs) and sets the cells to respond to CGs. Our work reveals that HCMV infection significantly induces the α3 isoform; guinea pig CMV (GPCMV) also induces α3, while MCMV infection does not induce mouse α3. We show that guinea pig CMV (GPCMV) is inhibited by CGs in vitro with a similar selectivity index to that of HCMV inhibition, although the EC_50_ and CC_50_ are higher and α3 induction is moderate. The activity of digitoxin against HCMV correlates with reduced α3 expression, and inhibition of HCMV replication with digitoxin analogs also correlates with the expression of the α3 isoform of the Na^+^/K^+^-ATPase. Intriguingly, the α3 isoform was localized in the nuclear compartment (while α1 was detected in the membrane fraction), and changes in its expression during infection and treatment with digitoxin were mainly limited to the nucleus. The α3 isoform colocalized with the promyelocytic leukemia protein (PML) in non-infected and HCMV-infected cells. At 24 h post-infection, the α3-PML-IE1 nuclear interaction was disrupted with digitoxin. In HCMV-infected digitoxin-treated cells, the mRNA levels of NF-κB, interferon-β (IFN-β), CXCL-10, IL-1 β, and IL-8 were reduced, suggesting that CGs target the PML-IE1 complex via the α3 isoform in the early stages of infection, resulting in HCMV inhibition.

## 2. Materials and Methods

### 2.1. Compounds

Digitoxin and ganciclovir (GCV) were purchased from Sigma Chemicals (St. Louis, MO, USA). The digitoxin analogs α-L-amicetose and tris-L-amicetose were previously reported [16,26,27]. All compounds were dissolved in dimethyl sulfoxide (DMSO) except for GCV, which was dissolved in distilled water.

### 2.2. Viruses

The HCMV pp28-luciferase Towne strain was constructed as previously reported [28]. This virus expresses luciferase under the control of the pp28 late promoter. Luciferase expression is strongly activated 48–72 h post-infection (hpi). This highly sensitive reporter system correlates with plaque reduction [28]. HCMV Towne (ATCC VR-977) and TB40 (ATCC VR-1578) were also used. The epithelial/endothelial cell-adapted TB40E/E virus was kindly provided by Dr. Laura Hertel, School of Medicine, University of California, San Francisco. MCMV and GPCMV strains were obtained from ATCC (VR-1399 and VR-682, respectively). HCMV TB40 was obtained from ATCC (VR-1578).

### 2.3. Cell Culture, Virus Infection, and Antiviral Assays

Human foreskin fibroblasts (HFFs) passage 12–16 (ATCC, CRL-2088) were grown in Dulbecco’s modified eagle medium (DMEM) containing 10% fetal bovine serum (FBS) (Gibco, Carlsbad, CA, USA) in a 5% CO_2_ incubator at 37 °C and used for infection with HCMV at a multiplicity of infection of 1 PFU/cell (MOI = 1), unless otherwise specified. Following a 90-min adsorption period, the media were removed, cells were washed with phosphate-buffered saline (PBS), and DMEM with 4% FBS-containing compounds was added to each well.

Infected, treated HFFs were collected at 72 hpi, and lysates were assayed for luciferase using a luciferase assay kit (Promega, Madison, WI, USA) on GloMax^®^-Multi+ Detection System (Promega) according to the manufacturer’s instructions.

Human retinal pigmented epithelial cells (ARPE-19) were purchased from ATCC (CRL-2302) and maintained in DMEM: F-12 medium (ATCC, 30-2006) containing 10% FBS. A total of 0.2 × 106 ARPE-19 cells were seeded in a 12-well plate. The following day, cells were infected with TB40 E/E (MOI = 3) for 90 min. The media were removed and replaced with the appropriate medium (Digitoxin 50 nM or GCV 5 µM). Samples were harvested at 24 and 72 h post-infection.

Mouse embryonic fibroblasts (MEFs, from Dr. Gordon Sanford, Johns Hopkins University School of Medicine) and GP lung fibroblasts (ATCC CCL-158) were used for infection with MCMV and GPCMV, respectively. For plaque assays, cells were seeded at 1.6 × 10^5^ cells per well in a 12-well plate and infected 24 h later with MCMV or GPCMV at 100 PFU/well. Following a 90-min adsorption period, the medium was aspirated, and a fresh medium containing digitoxin at the indicated nM concentrations and 0.5% of carboxymethyl-cellulose were added into duplicated wells. After incubation at 37 °C for 4 days, the overlay was removed, and the monolayer was stained with crystal violet. Plaques were counted microscopically under low power (40×). Drug effects were calculated as the percent reduction in plaque number in the presence of each drug concentration to the number observed in the absence of the drug.

### 2.4. Cytotoxicity Assay

The MTT assay was performed as per the manufacturer’s instructions (Sigma-Aldrich). Non-infected GP fibroblasts were treated with digitoxin for 4 days (same time points as the antiviral assay), and 20 μL/well of MTT [3-(4, 5-Dimethyl-2-thiazolyl)-2, 5-diphenyl-2 H-tetrazolium bromide], 5 mg/mL in PBS, was added to each well. After shaking at 150 rpm for 5 min, the plates were incubated at 37 °C for 2–3 h. The conversion of the yellow solution to dark blue formazan by mitochondrial dehydrogenases of living cells was quantified by measuring absorbance at 560 nm.

### 2.5. Quantitative Reverse Transcriptase PCR (qRT-PCR)

Total RNA was isolated from cultured cells using an RNeasy mini kit (Qiagen, Georgetown, MD, USA). RevertAid first-strand cDNA synthesis kit (Fermentas Life Sciences, Cromwell Park, MD, USA) was used to synthesize first-strand cDNA from total RNA using oligo (dT) primers. Synthesis of first-strand cDNA from an mRNA template was carried out at 42 °C for 1 h. The expression level of human, mouse, and GP ATPase isoforms and GP-encoded UL83 [29] was quantified using sequence-specific primers (Table 1) and real-time PCR with SYBR green (Fermentas Life Sciences, Cromwell Park, MD, USA), using the following conditions: initial denaturation at 95 °C for 10 min, followed by 95 °C for 15 s, 60 °C for 1 min for a total of 40 cycles, followed by melting curve analysis starting at 64 °C and ending at 95 °C, then a final hold at 4 °C. GP-GAPDH (glyceraldehyde-3-phosphate dehydrogenase) was used as an internal control. HCMV IE1 and MIEP-derived IE mRNA levels were measured [30,31]. The following antiviral genes were measured during infection and treatment with digitoxin: NF-κB, IFN-β, IL-1β, CXCL-10, and IL-8 (Table 2). Human-GAPDH was used as an internal control.

### 2.6. SDS-Polyacrylamide Gel Electrophoresis and Immunoblot Analysis

Cell lysates containing an equivalent amount of proteins were mixed with an equal volume of sample buffer (125 mM Tris-HCL, pH 6.8, 4% SDS, 20% glycerol, and 5% *β*-mercaptoethanol) and boiled at 100 °C for 10 min. Denatured proteins were resolved in tris-glycine polyacrylamide gels (10–12%) and transferred to polyvinylidine difluoride (PVDF) membranes (Bio-Rad Laboratories, Hercules, CA, USA) by electroblotting. Membranes were incubated in blocking solution [5% non-fat dry milk and 0.1% Tween-20 in PBS (PBST)] for 1 h, washed three times with PBST, and incubated with appropriately diluted primary antibodies at 4 °C overnight. Membranes were washed with PBST and incubated with horseradish peroxidase-conjugated secondary antibodies in PBST for 1 h at room temperature. Following washing with PBST, protein bands were visualized by chemiluminescence using SuperSignal West Dura and Pico reagents (Pierce Chemical, Rockford, IL, USA). The anti-Na^+^/K^+^-ATPase α3 mouse monoclonal antibody (XVIF9-G10) and α1 rabbit monoclonal antibody (AB76020) were from Abcam (Cambridge, MA, USA). The HCMV IE1/2 antibody was from Millipore Sigma (MAB810R, Billerica, MA, USA). The β-actin monoclonal antibody was from Sigma-Aldrich (A3853, St. Louis, MO, USA).

### 2.7. Cellular Localization of α3

Two million HFFs were plated on a six-well plate. The following day, cells were infected with HCMV-Towne and treated with digitoxin or GCV. Cells were fractionated at 24 and 72 h using the Qproteome Cell Compartment kit (Qiagen, Hilden, Germany) following the manufacturer’s instructions. Briefly, HFFs were washed and treated with specific buffers to obtain the cytoplasmic and nuclear fractions, precipitated with acetone and quantified using a BCA assay kit (Thermo Scientific, Rockford, IL, USA). An equal amount of protein was analyzed by immunoblotting, and cytoplasmic and nuclear fractions were confirmed by probing for GAPDH (Santa Cruz Inc., sc-47724, Dallas, TX, USA) and histone H3 (Cell Signaling Technology, 4499S, Beverly, MA, USA) as internal controls, respectively.

### 2.8. NLS Analysis of the α3 Isoform

Linear motif analysis through multiple software packages, including eukaryotic linear motif (ELM) [32], NovoPro [33], and NLSExplorer (doi.org/10.1101/2024.08.10.606103), were used to predict nuclear localization signals (NLS) in α3. Sequences were identified through hidden Markov models and highlighted in the alignment of the three major isoforms of α3, with the amino acid contribution determined by NLSExplorer (doi.org/10.1101/2024.08.10.606103).

### 2.9. Immunofluorescence Assay

A total of 0.5 × 10^6^ HFFs were seeded on chamber slides. The following day, cells were infected with HCMV-TB40 (MOI = 1) for 90 min and then treated with digitoxin (50 nM) or GCV (5 μM). After 24 h, supernatants were discarded, and cells were washed thrice with cold 1× PBS. Cells were fixed with fresh 4% paraformaldehyde for 10 min on ice, permeabilized with 0.1% Triton-100 PBS for 15 min and incubated with 10% goat serum for 45 min at room temperature. The following primary antibodies were added in 10% goat serum in PBST overnight at 4 °C: mouse anti-ATPase1 α3 monoclonal antibody (XVIF9-G10) (1:200, MA3-915, Thermo Fisher), rabbit anti-PML (E6S9L, 1:200, Cell Signaling, 69789, Beverly, MA, USA), mouse anti-CMV IE1/2 (1:200, MAB810R, Millipore Sigma), and rabbit anti-ATPase1 α3 monoclonal antibody (1:200, BLP-NP003, Allomone Labs). The primary antibody solution was removed, and cells were washed thrice with PBST, 5 min for each wash. A secondary antibody was added: goat anti-mouse IgG Fab2 Alexa 555 (1:500, Cell Signaling 4409S), goat anti-rabbit IgG (H + L) Alexa 488 (1:500, Cell Signaling 4412S), for 1 h at room temperature under dark conditions, then shaking was performed at 60 rpm for 20 min at room temperature. The secondary antibody solution was removed, and cells were washed thrice with PBST, 5 min each wash. Slides were dried at room temperature. One drop of DAPI (4′,6-diamidino-2-phenylindole, Santa Cruz, SC-24941) was added into each chamber, then a cover slip was applied, and the edge was sealed with nail polish. Images were taken with a Zeiss Confocal microscope under a water 40× object lens.

Images were divided into nine equal sub-images and analyzed for Pearson’s colocalization coefficient [34] using the JACoP plugin for ImageJ, v2.1.4 [35]. Sub-images lacking pixels from one fluorophore were removed from the analysis. Graphs were plotted and analyzed using the GraphPad Prism 10.2 software.

### 2.10. Nuclear Coimmunoprecipitation and Immunoblotting

Two million HFFs were plated in 100 mm petri dish (three dishes for treatment), infected the following day with HCMV TB40 (MOI of 1), and treated with digitoxin (50 nM) or GCV (5 µM) for 24 h. Cells were harvested and spun down, the supernatant was removed, and cell pellets were stored at −80 °C. Nuclei were isolated using a nuclear complex Co-IP kit (Active Motif, #54001, Carlsbad, CA, USA). Briefly, cell pellets were resuspended in 500 µL 1× hypotonic buffer and incubated for 15 min on ice. A detergent was added, and the suspension was centrifuged for 30 s at 14,000× *g* in a microcentrifuge pre-cooled at 4 °C. The cytoplasmic fraction was discarded, and the nuclear fraction was kept on ice. The nuclear pellet was resuspended in a complete digestion buffer, and an enzymatic shearing cocktail was added. The suspension was vortexed gently and incubated for 90 min at 4 °C. The reaction was stopped with 2 µL 0.5 M EDTA followed by centrifugation for 10 min at 14,000× *g* in a microcentrifuge pre-cooled at 4 °C. The concentration of nuclear proteins was quantified using the BCA method. A total of 200 µg of extracted nuclear protein and 3 µg of antibody in 500 µL IP incubation buffer was incubated overnight at 4 °C on a rotator. Antibody binding beads (Protein A or G) were added to the antibody/extract mixture and incubated for 90 min at 4 °C. An IP wash buffer was added twice to each tube, mixed, centrifuged, and the supernatant was removed. The bead pellet was resuspended in 2× reducing loading buffer, and samples were boiled at 95–100 °C for 5 min. After the samples were cooled, they were briefly spun down, and a western blot analysis was performed. A total of 1% of the nuclear extract used for IP was loaded into the gel as “input”. The following antibodies were used: PML (E-11, Cat# sc-377390, Santa Cruz, Dallas, TX, USA) for PML pull-down, PML (Cat#A301-167A, Bethyl Laboratories, Montgomery, TX, USA) for western blot (1:1000), ATPase1 α3 (H-4, Cat#SC-365744, Santa Cruz, Dallas, TX, USA) for α3 pull-down, ATPase1 α3 (Cat#MA3-915, Invitrogen, Waltham, MA, USA, 1:1000) for WB, anti-CMV IE1/2 (clone 8B1.2, Cat#MAB810R, Millipore Sigma, Billerica, MA, USA, 1:1000) for western blot, Histone H3 (D1H2, Cat# 4499s, Cell signaling, Beverly, MA, USA, 1:3000) for western blot, anti-mouse IgG HRP, (Cat#7076s, Cell signaling, USA, 1:3000), and anti-Rabbit IgG HRP (Cat#7074s, Cell signaling 1:3000).

### 2.11. Statistical Analysis

The EC_50_ and CC_50_ values were calculated using the GraphPad Prism software v10.4.1 using the nonlinear curve fitting and the exponential form of the median effect equation, where percent inhibition = 1/[1 + (CC_50_ or EC_50_/drug concentration) *m*], with *m* reflecting the slope of the concentration–response curve. All statistical analyses were performed using the GraphPad Prism software. Individual points represent the mean ± SD (n ≥ 3). A single factor ANOVA was used to determine the significance of comparing groups, and * represents *p* ≤ 0.05, ** represents *p* ≤ 0.01, and *** represents *p* ≤ 0.001.

## 3. Results

### 3.1. α3 mRNA Is Induced in GPCMV and HCMV-Infected Cells but Not in MCMV-Infected Cells

**Figure 1:**
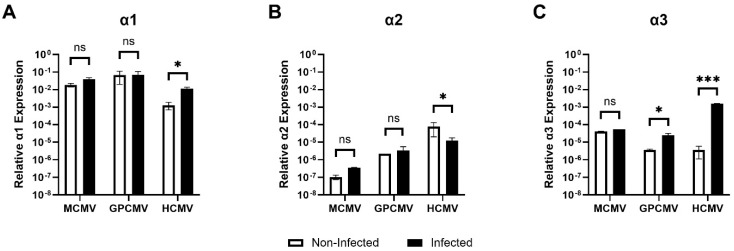
Changes in α1 (**A**), α2 (**B**), and α3 (**C**) mRNA levels in MCMV-, GPCMV-, and HCMV-infected cells. MEFs, GP fibroblasts, and HFFs were infected with MCMV, GPCMV, and HCMV (MOI 1 PFU/well), respectively. At 72 hpi, RNA was isolated from infected and non-infected cells, and the level of α isoforms was measured by qRT-PCR. The experiment was performed in triplicates and repeated thrice. * represents *p* ≤ 0.05, and *** represents *p* ≤ 0.001. ns not significant.

We tested whether different CMVs induce the expression of the α isoforms of the Na^+^/K^+^-ATPase. MEFs, GP fibroblasts, and HFFs were infected with MCMV, GPCMV, and HCMV, respectively, at a multiplicity of infection (MOI) of 1 plaque-forming unit (PFU)/cell. At 72 hpi, mRNA was isolated from infected and non-infected cells and the level of α isoforms was measured by quantitative RT-PCR (qRT-PCR, Figure 1). While no significant changes were observed in the levels of α1/α2/α3 isoforms between MCMV-infected and non-infected MEFs (Figure 1A–C), induction of α3 was observed in GPCMV-infected fibroblasts (Figure 1C). A significant upregulation of human α3 was detected in HCMV-infected HFFs (Figure 1C). In contrast, the changes in α1 or α2 transcripts were modest (Figure 1A,B).

### 3.2. Inhibition of GPCMV Replication with Digitoxin Correlates with Reduced α3 Expression

We, and others, have reported on the inhibition of HCMV replication with CGs and their lack of activity against MCMV [9,11,15,18]. At 30–50 nM concentrations, digitoxin inhibited HCMV replication to a similar level as GCV (5 µM), based on several antiviral assays, including pp28-luciferase activity, virus yield (by real-time PCR), and plaque reduction. Substitution of the sugar type and the sugar length in digitoxin analogs resulted in differential selectivity against HCMV [16]. The α-L-rhamnose and α-L-amicetose isomers had improved anti-HCMV activity compared to the enantiomeric α-D isomers and natural β-D-isomers of digitoxin. Within each stereoisomer, there was an inverse correlation between the sugar length and HCMV inhibition: the longer the oligosaccharide chain, the less effective the compound was against HCMV replication.

**Figure 2:**
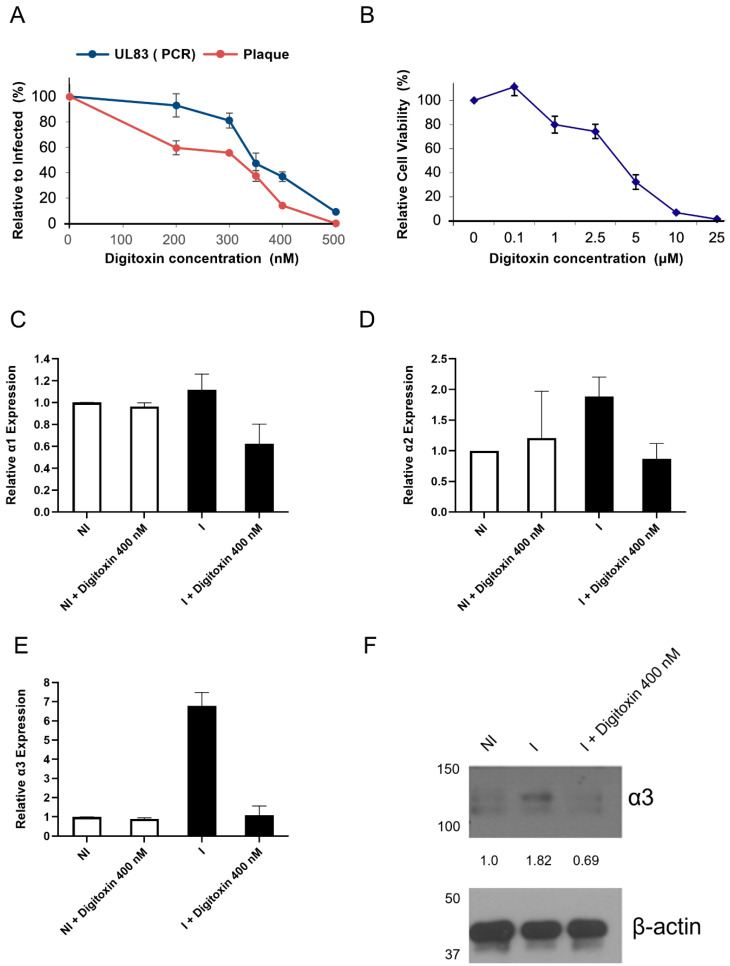
GPCMV inhibition and changes in the mRNA of α isoforms in non-infected and GPCMV-infected GP fibroblasts treated with digitoxin. (**A**) GPCMV replication was measured by plaque assay using 100 PFU/well (red) and DNA replication of UL83 (blue) at 4 days post-infection. (**B**) Relative cell viability was measured by MTT assay in non-infected GP fibroblasts at the same time point. All experiments were repeated thrice; the represented values are the mean ± SD. (**C**–**E**) GP fibroblasts were treated with digitoxin, infected with GPCMV, and treated with digitoxin. After 4 days, the mRNA of α1 (**C**), α2 (**D**), and α3 (**E**) was measured in non-infected digitoxin-treated cells and GPCMV-infected digitoxin-treated cells. (**F**) Western blot measured the level of α3 protein in non-infected (NI), infected (I), and I+ digitoxin-treated cells.

To evaluate a correlation between species-specific CMV inhibition and the changes in the induction of the isoforms of the Na^+^/K^+^-ATPase pump, we tested the activity of CGs against GPCMV using a plaque assay and UL83 PCR. GP fibroblasts were infected with GPCMV (100 PFU/well), treated with digitoxin, and, after four days, plaques were counted. UL83 DNA was measured in infected (MOI 0.5) and infected–treated cells. The EC_50_ of digitoxin based on plaque assay and viral DNA replication was 350 nM ± 20 (Figure 2A). Cell viability was measured in non-infected GP fibroblasts at the same time point. The CC_50_ was 3.6 µM ± 0.2, resulting in a selectivity index (CC_50_/EC_50_) of 11 ± 0.09 (Figure 2B).

The expression level of α1, α2, and α3 was measured in non-infected GP fibroblasts, GPCMV-infected, and infected–treated cells. In non-infected cells treated with digitoxin, α1, α2, and α3 mRNA levels were unchanged (Figure 2C–E). Infection and digitoxin treatment did not change the expression of α1 or α2 (Figure 2C,D), but α3 was induced in infected cells (Figure 2E) and reduced with the digitoxin treatment. A western blot analysis similarly showed the induction of α3 protein during infection and its reduction with digitoxin (Figure 2E).

### 3.3. Levels of α3 mRNA and Protein Are Reduced in HCMV-Infected Digitoxin-Treated Cells

**Figure 3:**
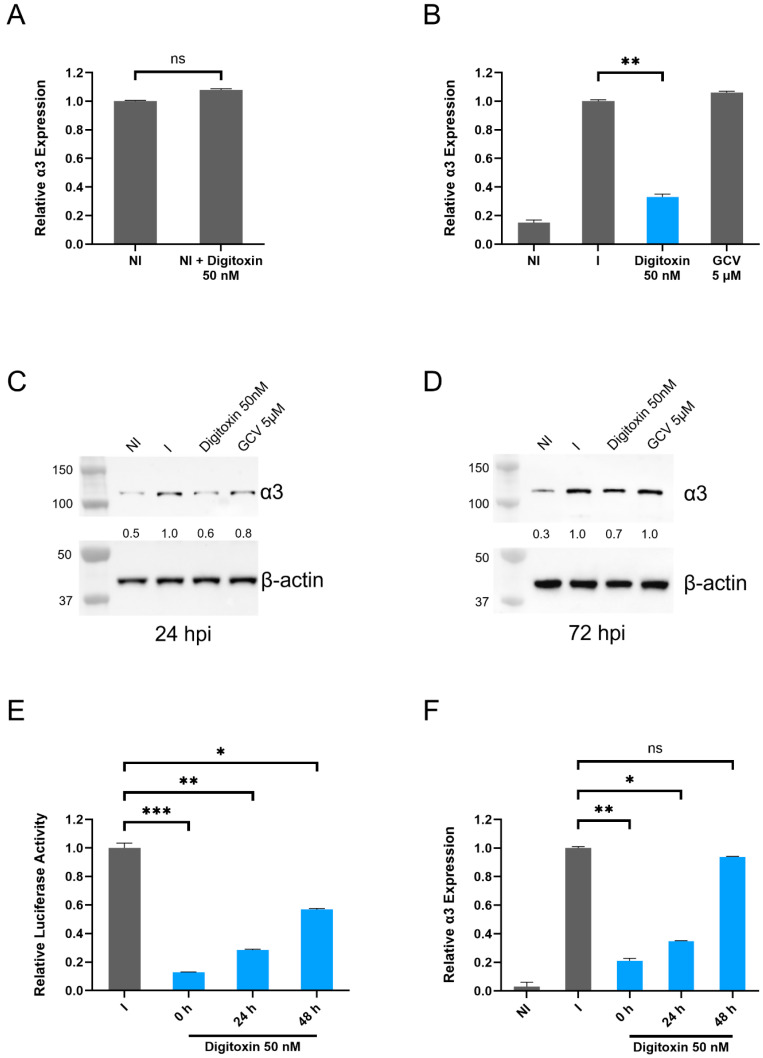
Changes in α3 expression in HCMV-infected cells treated with digitoxin. (**A**) The level of α3 mRNA was measured in non-infected (NI) HFFs treated with digitoxin and (**B**) in HCMV-Towne-infected cells (I) at 72 hpi. (**C**,**D**) The expression of α3 protein was measured in non-infected, HCMV-infected HFFs (TB40, MOI—1 PFU/cell), and infected–treated cells at 24 and 72 hpi. The experiment was performed twice; representative immunoblots are presented. Numbers adjacent to the immunoblots are kDa values. (**E**,**F**) The effect of the time of addition of digitoxin (0, 24, or 48 hpi) was tested by pp28-luciferase activity and α3 mRNA level in HFFs infected with pp28- Towne. * represents *p* ≤ 0.05, ** represents *p* ≤ 0.01, and *** represents *p* ≤ 0.001. ns not significant.

We next tested whether HCMV inhibition by digitoxin was associated with changes in α3 expression. At 72 h, in non-infected HFFs, digitoxin (**1**) (Supplementary Figure S1) did not have an effect on α3 expression (Figure 3A), but, in HCMV-infected HFFs, virus inhibition with digitoxin correlated with decreased expression of α3, an effect that was not observed in GCV-treated infected cells (Figure 3B). Digitoxin (**1**) reduced the expression of α3 protein at both 24 and 72 hpi (Figure 3C,D). A similar effect on α3 protein level was observed in TB40E/E-infected ARPE-19 cells (Supplementary Figure S2). The addition of digitoxin at 24 and 48 hpi resulted in reduced activity against HCMV replication and a decreased effect on α3 expression (Figure 3E,F).

### 3.4. Concentration-Dependent Decrease in α3 Expression in HCMV-Infected HFFs Treated with Digitoxin Analogs

**Figure 4:**
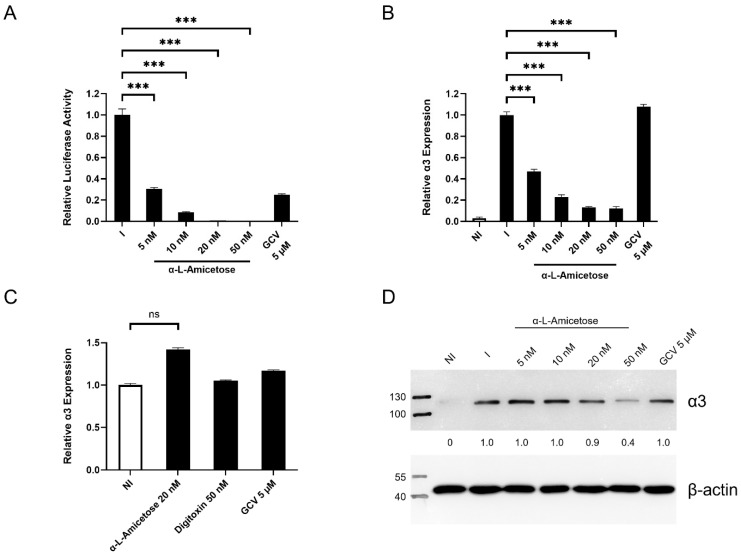
Concentration-dependent decrease in α3 expression in HCMV-infected HFFs treated with α-L-amicetose. (**A**) HFFs were infected with pp28-luciferase and treated with different concentrations of α-L-amicetose or GCV (5 µM). At 72 hpi, lysates were collected and assayed using a luciferase assay kit (Promega, Madison, WI, USA). (**B**) The relative α3 mRNA level was measured at 72 hpi. (**C**) The relative α3 mRNA level was measured at 72 h in non-infected cells. (**D**) The expression of α3 protein in NI, I, and I-treated cells was measured by western blot at 72 hpi with HCMV TB40. The experiment was performed thrice, and the best representative image is depicted. Numbers adjacent to the immunoblots are kDa. *** represents *p* ≤ 0.001. ns not significant.

We evaluated the activity correlation of synthetic digitoxin analogs **2** and **3** (Supplementary Figure S1) and changes in α3 expression. The α-L-amicetose analog **3** inhibited HCMV pp28-luciferase activity in a dose–response manner and decreased the expression of α3 in HCMV-infected HFFs (Figure 4A,B). There was no difference in α3 expression in non-infected HFFs treated with compounds **1** and **2**, suggesting a role for viral proteins in this activity (Figure 4C). The changes in α3 transcripts and protein were dose-dependent and correlated with HCMV inhibition (Figure 4A,B,D).

**Figure 5:**
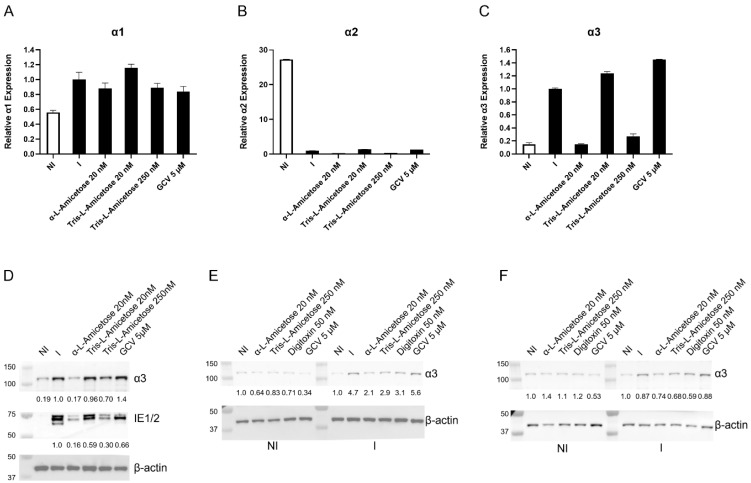
Effect of sugar length on the levels of α1, α2, and α3 mRNA (**A**–**C**) and α3 protein (**D**). Changes in mRNA of α1 (**A**), α2 (**B**), and α3 (**C**) were measured after 72 h in non-infected (NI) HFFs and in infected (I) cells treated with the indicated concentrations of α-L-amicetose and tris-L-amicetose. Western blot analysis was performed for IE1/2 and α3 at 72 hpi (**D**). The experiment was performed twice, and the best representative images are depicted. Numbers adjacent to the immunoblots are kDa. (**E**,**F**) Changes in α3 levels in non-infected (NI) and HCMV-infected (I) HFFs treated with digitoxin analogs at a concentration of full virus suppression (α–L amicetose and tris-L amicetose) at 24 h (**E**) and 72 h (**F**). The experiment was performed twice, and the best representative images are depicted.

Similarly, α3 levels correlated with the length of sugar attached to the cardiac glycoside core (Figure 5). While no significant difference was observed in α1 or α2 mRNA during infection and compound treatment (Figure 5A,B), the longer the sugar chain (i.e., compounds **2** vs. **3**), the weaker the effect on α3 mRNA and protein expression (Figure 5C,D). Comparing digitoxin to two analogs at concentrations of near complete HCMV inhibition, α-L-amicetose **2** (20 nM) and tris-L-amicetose **3** (250 nM) similarly showed no difference in α3 level in non-infected, treated cells but reduced α3 levels in HCMV-infected cells at both 24 h and 72 h post-infection (Figure 5E,F).

### 3.5. α3 Is Localized in the Nuclear Compartment and Interacts with PML and IE1 During Infection

**Figure 6:**
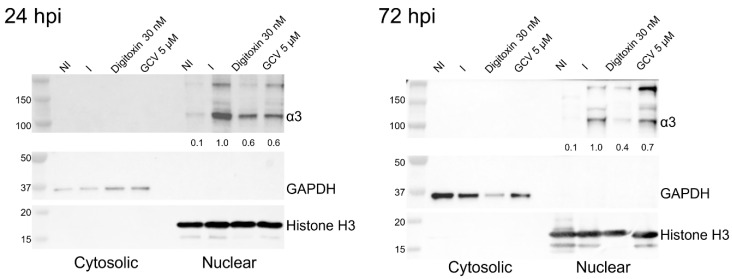
Localization of the α3 isoform in the nuclear fraction. The level of α3 was measured in non-infected and HCMV-infected cells treated with digitoxin or GCV at 24 and 72 hpi. GAPDH and histone H3 were used as controls for the cytosolic and nuclear fractions, respectively. The experiment was performed thrice, and the best representative images are depicted. Numbers adjacent to the immunoblots are kDa.

Since we observed significant changes in the level of α3 during HCMV infection and based on the timing of HCMV inhibition by CGs, we asked in which cell compartment these changes occurred. Intriguingly, at both 24 and 72 hpi, α3 was significantly induced in the nuclear fraction (Figure 6), while α1 was mainly in the membrane fraction (Supplemental Figure S3). Digitoxin, but not GCV, reduced the level of nuclear α3 (Figure 6).

**Figure 7:**
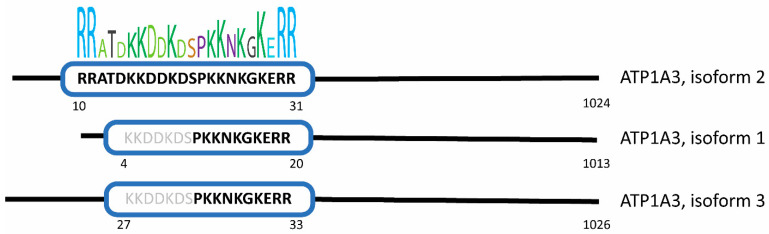
Prediction of NLS in the α3 isoforms. The sequence of each isoform was processed through multiple software packages to predict if and where the sequence of α3 aligned with previously identified NLS motifs. NLSExplorer further depicted the hydrophobicity and frequency of each α3 amino acid in the predicted NLS with consensus and other known NLS motifs.

Since the nuclear localization of α3 was unexpected, we asked whether the protein’s amino acid sequence shows a strong probability to contain a nuclear localization signal. Using the eukaryotic linear motif (ELM), a monopartite and bipartite version of the classical charged NLS were predicted (probability 2.59 × 10^−4^ and 1.28 × 10^−3^, respectively) near the N-terminus of α3 (amino acids 10–31) in isoform 2 [32]. Sequence similarity and motif analysis of α3 isoforms 1 and 3 also strongly indicate the presence of the monopartite NLS at the N-terminus (isoform 1, amino acids 4–20, and isoform 3, amino acids 27–33), Figure 7.

In an immunofluorescence assay (IFA), α3 was primarily detected in the nuclear fraction of infected cells, showed a speckled pattern, and colocalized with IE1 (Supplemental Figure S4). We, therefore, tested whether α3 colocalized with the promyelocytic protein (PML). A predominantly nuclear protein, PML forms heterogeneous multiprotein subnuclear structures (nuclear bodies—NBs; PML oncogenic domains—POD; ND10 bodies) with diverse functions related to the control of gene expression. At least 50 different proteins have been shown to localize to PML NBs, constitutively or transiently, to mediate various processes, including the response to DNA damage, cell cycle control, antiviral response, and apoptosis [36]. PML undergoes SUMOylation, phosphorylation, ubiquitination, and acetylation in response to cellular stress [37]. PML is a key regulator of cytokine responses and innate immunity [38,39]. Specific PML isoforms are positive regulators of interferon (IFN) synthesis. PML proteins may induce some IFN-stimulated genes (ISGs) [40,41].

**Figure 8:**
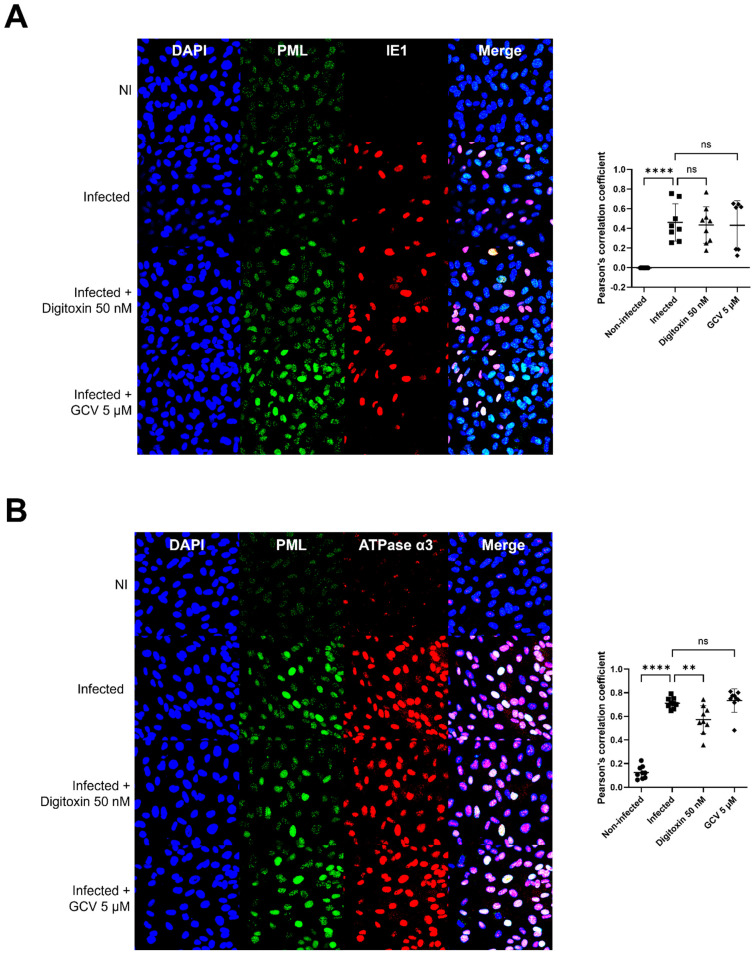
PML-α3 and PML-IE1 colocalization. IFA was performed at 4 h in non-infected, infected, and infected-compound-treated cells. Co-staining was performed with mouse anti-CMV IE1/2 and rabbit anti-PML (**A**), rabbit anti-PML, and mouse anti-α3 (**B**). The experiment was repeated thrice, and the best images are shown. Quantification of colocalization of PML and IE1 (A, upper) and PML and α3 (B, lower) is shown using Pearson’s correlation coefficients. Data represent an average of nine measurements with SD. Significance levels were calculated using one-way ANOVA followed by Dunnett’s multiple comparisons test (**** *p* <0.0001, ** *p* <0.01, ns not significant).

In non-infected HFFs, PML showed speckled staining. PML was dispersed and colocalized with IE1 at 4 hpi with HCMV TB40 (MOI 3), and there was no significant difference with digitoxin treatment (Figure 8A). There was significant colocalization of PML and α3 which was reduced with digitoxin treatment, but not with GCV (Figure 8B).

**Figure 9:**
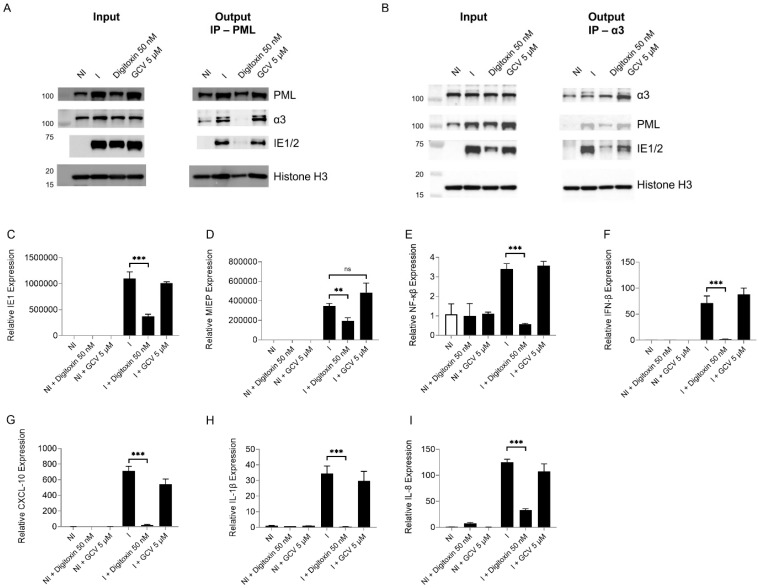
The nuclear interaction of α3-PML-IE1 is disrupted by digitoxin in HCMV-infected cells, and antiviral response genes are reduced. (**A**) Immunoprecipitation (IP) of PML was performed in HCMV-infected (MOI of 1), digitoxin (50 nM)-treated, or GCV (5 µM) cells at 24 hpi. IE1 and α3 were probed for analysis of their interaction with PML. The left panel shows the profile of different proteins in the nucleus (input), used for IP. (**B**) Reverse IP of α3 was performed in HCMV-infected digitoxin-treated cells at 24 hpi. IE1 and PML were probed to measure the status of α3-IE1-PML complex. The left panel shows the input used for the IP. (**C**–**I**) mRNA levels of IE1, MIEP-derived IE, IFN-β, CXCL-10, IL-1β, and IL-8 were measured in NI and I, digitoxin- and GCV-treated cells at 24 hpi. The experiments were performed in triplicates and repeated twice. ** represents *p* ≤ 0.01, and *** represents *p* ≤ 0.001. ns not significant.

Co-immunoprecipitation assays performed at 24 hpi with TB40 (MOI 1) revealed an interaction between PML, α3, and IE1 which was reduced with digitoxin (Figure 9A,B). Finally, we measured the mRNA level of IE1, major immediate-early promoter (MIEP)-derived IE, and antiviral cytokines during infection and digitoxin treatment. At 24 hpi, HCMV induced IE1, MIEP, interferon-β (IFN- β), CXCL-10, IL-1 β, and IL-8. Treatment with digitoxin reduced IE1 and MIEP levels. It abolished the IFN-β and cytokine response (Figure 9C–I). These data suggest a mechanism of HCMV inhibition involving proteins that play a role in antiviral immunity.

## 4. Discussion

We report that HCMV infection of HFFs results in significant induction of the α3 isoform of the Na^+^/K^+^-ATPase, that the anti-HCMV activity of digitoxin analogs (**2**) and (**3**) correlates with decreased expression of the α3 isoform, and that GPCMV induces α3 and is also inhibited with digitoxin (**1**).

Overexpression of the α3 isoform has been reported in colon and pancreatic cancer cells [42,43], and we show that this isoform is upregulated in HCMV-infected HFFs. In non-infected cells, the mRNA levels of the three isoforms (α1, α2, α3) were low. HCMV induced α3 transcripts by 1000-fold and α1 by 10-fold, while α2 transcripts decreased by 10-fold. The anti-HCMV activities of CGs correlated with changes in the expression of α3, an effect that was dose-dependent and time-dependent.

Human tumor cells commonly express both α1 and α3 isoforms, but rodent tumor cell lines only express α1 [44]. A preferential binding of ouabain to the α3 isoform over α1 and α2 has been described [45]. A decrease in α1 and an increase in α3 isoform has been found in colon tissues when their normal phenotype changed to a malignant one [42], suggesting that tumor tissue is a more sensitive target than normal tissue to CGs. The CG, oleandrin, inhibited human pancreatic cancer cell growth but not rodent tumor cell proliferation, an activity that correlated with the relative expression of α3. The higher α3 expression relative to α1, the more sensitive the cell was to oleandrin [43].

Earlier studies suggest that an α2-selective CG could result from sugar modification because the structural differences in these isoforms are primarily in the carbohydrate-binding loops [46]. The role of the sugar type and sugar length attached to the CG aglycone core has been tested in human lung cancer cells [27]. Cardiac glycoside analogs glycosylated with enantiomeric sugars α-D-rhamnose and α-D-amicetose exhibited reduced apoptosis compared to α-L-rhamnose and α-L-amicetose. Both α-L-rhamnose and α-L-amicetose induced cell death in concentration-dependent and sugar-chain length-dependent manners. The α-L-rhamnose and α-L-amicetose showed potency at least 10 times stronger than that of the corresponding di- and tri-saccharide analogs [27]. It is unclear whether the differential activity of the stereo- and oligo-isomers of digitoxin is directly related to changes in expression or activity of specific α isoforms. Our data indicate a direct correlation between the sugar type and length, α3 expression, and HCMV inhibition.

Although classically a plasma membrane pump, intriguingly, α3 localized to the nucleus upon HCMV infection, suggesting a unique and novel mechanism of action that may be independent of ion exchange. Indeed, α3 harbors an NLS. Studies in lung and colonic cancer cell lines reveal that the intracellular location of α3 differs between human cancer and normal cells. While localized near the cytoplasmic membrane in normal human colon and lung epithelia, α3 expression is perinuclear in the respective cancer cells [47]. Our IFA shows that α3 and PML colocalize in the nucleus. CGs reportedly induce PML via a post-transcriptional mechanism [48]. Inhibition of pump activity correlates with the EC_50_ of PML NB formation. Interestingly, in murine cells, CGs do not induce PML formation. Although, this could be attributed to the rodent α1 isoform being insensitive to binding and modulation by CGs, based on two amino acid differences from the human pump [2]. Our data indicate that α3 (not induced in MCMV-infected cells) may also play a role in PML activation.

PML-NBs contain hundreds of proteins with activities in a variety of cellular pathways [36,49,50,51,52,53,54,55,56,57,58]. PML-NBs are factories dedicated to the SUMOylation of many proteins, including PML itself, to regulate their stability and/or activity in the nucleus [59]. The association of the protein SUMO modification pathway with PML-NBs is likely implicated directly and/or indirectly in their intrinsic antiviral defense activity against viruses [38,60]. IE1 directly interacts with the PML coiled-coil domain and disrupts NB foci by inducing a loss of PML SUMOylation [61]. Via binding to PML, IE1 was suggested to compromise both the intrinsic antiviral defense mechanisms and the classic innate immune responses. Moreover, PML targeting by IE1 is thought to promote HCMV replication, partially by inhibiting PML-dependent IFN and ISG expression triggered by viral infection [61]. However, a recent report suggests that disruption of PML bodies results in induction rather than inhibition of antiviral gene expression [62]. A mutant IE1 that abolished PML binding without affecting other activities did not interact with PML, suggesting that the interaction with PML may not be central to the function of IE1 and providing evidence for an antiviral rather than proviral effects of PML body disruption [62]. The IE1-PML interaction was associated with enhanced rather than reduced expression of cytokines and interferon stimulatory genes (ISGs), suggesting that disruption of PML by IE1 contributes to the antiviral and proinflammatory response during HCMV infection. We show that α3 interacts with IE1 and PML, and that digitoxin treatment reduces this interaction and decreases the antiviral response. In infected digitoxin-treated cells, IFN-β, CXCL-10, IL-1β, and IL-8 levels are significantly reduced, suggesting that nuclear α3 modulates IE1-PML early during infection.

The expression of the human neuronal α3 is regulated by the activity of the Sp1 and NF-Y transcription factors [63]. HCMV induces Sp1 mRNA expression and protein level, which, in turn, induces NF-kB [64]. Therefore, α3 may also play a role in the induction of nuclear transcription factors, inhibiting virus replication. Indeed, the direct and indirect targets of CGs can be nuclear receptors [65]. Other transcription factors that interact with IE1 (e.g., CEBP, E2F1-5, SP1) may induce α3 [66], and HCMV proteins may interact with α3 to induce a specific transcription factor signature.

Molecular events before the onset of HCMV DNA replication have been largely neglected in antiviral approaches [66]. HCMV activates or inhibits numerous signaling pathways, many exhibiting both pro- and antiviral potential. The initial NF-kB response to HCMV infection facilitates IE expression via binding sites in the proximal enhancer of the MIEP. While this response may benefit HCMV replication, it adversely affects the virus. NF-kB stimulates the transcription of numerous cytokine and chemokine genes. Some of these genes encode antiviral proteins, including type I IFNs. In addition to their primary target, cardiac glycosides reportedly activate or inhibit multiple signaling pathways, such as inhibition of DNA topoisomerase II, NF-κB-mediated pathways, and interleukin (IL)-8 production [13,67]. Digitoxin may prevent immune-related events triggered by HCMV that are needed to initiate efficient virus replication and are not inhibited by early and late inhibitors. The nature of α3-PML-IE1 interactions and activities needs further investigation. However, reducing their levels in HCMV-infected cells may provide CGs with a novel antiviral mechanism that abrogates IE1’s antagonistic activity towards PML.

## Figures and Tables

**Table 1:** Primers used for qRT-PCR of the α isoforms in human, mouse, and GP fibroblasts.

Gene	Primer Sequence
Human α1—Fwd	5′-CCAAGCTGCTACAGAAGAGGAACC-3′
Human α1—Rev	5′-TTGAAGGATTCCATGATCTTTGAAC-3′
Human α2—Fwd	5′-CGCTCGACAAGGAGATGCAAGA-3′
Human α2—Rev	5′-CCGAGGAAACTTTCCAGATGGC-3′
Human α3—Fwd	5′-TTC GGG GGC TTC TCC ATC CT-3′
Human α3—Rev	5’-GCA CTC GGT CTC CAC CCT TGA-3’
Mouse α1—Fwd	5’-CCGTGGATAACCTCTGCTTCGT-3’
Mouse α1—Rev	5’-CGCTGTGATTGGATGGTCTCCT-3’
Mouse α2—Fwd	5’-CTGTCCTTGGATGAGCTGGGC-3’
Mouse α2—Rev	5’-ACTTGACCCACTCAGGAGTTGTGG-3’
Mouse α3—Fwd	5’-GGTGTGGGTATCATCTCTGAGG-3’
Mouse α3—Rev	5’-CGTCAATCTGCTCCGAGGTGAA-3’
GP α1—Fwd	5’-CAGGGATATGGATGAACTGAAGAA-3’
GP α1—Rev	5’-GTGTGAGGGAATTAGGACCATCTC-3’
GP α3—Fwd	5’-GGAGTGGGCATCATTTCTGAGG-3’
GP α3—Rev	5′-CGTCGATTTGCTCTGAGGTGAA-3′
GP α2- Fwd	5′-CTGTCCTTGGATGAGCTGGGT-3′
GP and mouse α2—Rev	5′-ACTTGACCCACTCAGGAGTTGTGG-3′
UL83 F6	5′-CGACGACGACGATGACGAAAAC-3′
UL83 B11	5′-TCCTCGGTCTCAACGAAGGGTC-3′
GP-GAPDH—Fwd	5′-GGGCAAGGTCATCCCAGAG-3′
GP-GAPDH—Rev	5′-TGGAAGAATGGCTGTCACTGTT-3′

**Table 2:** Primers for quantitative RT-PCR of HCMV IE1, MIEP, and human antiviral response genes.

Gene	Primer Sequence
HCMV IE1—Fwd	5′-CTTAATACAAGCCATCCACA-3′
HCMV IE1—Rev	5′-TAGATAAGGTTCATGAGCCT-3′
HCMV MIEP-derived IE—Fwd	5′-TTGACCTCCATAGAAGACAC-3′
HCMV MIEP-derived IE—Rev	5′-AGGACTCCATCGTGTCAAGG-3′
Human CXCL-10—Fwd	5′-TTACTGAAAGCAGTTAGCAAGGAA-3’
Human CXCL-10—Rev	5’-AGCTGATTTGGTGACCATCATTG-3’
Human IFN-β—Fwd	5’-GATTCATCTAGCACTGGCTGG-3’
Human IFN-β—Rev	5’-CTTCAGGTAATGCAGAATCC-3’
Human IL-8—Fwd	5’-TGCAGCTCTGTGTGAAGGTGCAGT-3’
Human IL-1β—Fwd	5’-GCTCGCCAGTGAAATGATGGCTT-3’
Human IL-1β—Rev	5’-CAGAGGTCCAGGTCCTGGAAGG -3’
Human IL-8—Rev	5’-CAGTGTGGTCCACTCTCAATCACTC-3’
Human GAPDH—Fwd	5’-TTGGTATCGTGGAAGGACTC-3’
Human GAPDH—Rev	5’-ACAGTCTTCTGGGTGGCAGT-3’

## Data Availability

The data that support the findings of this study are available on request from the corresponding author.

## Supplementary Materials

The following supporting information can be downloaded at: https://www.mdpi.com/article/10.3390/v17030398/s1, Supplementary Figure S1: Structure of the Cardiac Glycosides. (1) Digitoxin, a glycosylated natural product consisting of an aglycon (digitoxigenin in blue) and β-linked 1,4-D-digitoxose trisaccharide. (2) Digitoxigenin α-L-amicetoside, a synthetic cardiac glycoside analog consisting of a glycosylated digitoxigenin (blue) with a 3-deoxy-L-sugar (i.e., α-linked L-amicetose). (3) Digitoxigenin tris-a-L-amicetoside, a synthetic cardiac glycoside analog consisting of a glycosylated digitoxigenin (blue) with a 3-deoxy-L-sugar trisaccharide (i.e., tris-a-linked L-amicetose). Supplementary Figure S2: α3 expression in TB40E/E-infected ARPE-19 cells. The expression of α3 protein was measured in non-infected, HCMV-infected HFFs (TB40E/E, MOI—3 PFU/cell), and infected treated cells at 24 and 72 hpi. The experiment was performed twice; representative immunoblots are presented. Numbers adjacent to the immunoblots are kDa values. Supplementary Figure S3: localization of the α1 and α3 isoforms of the Na^+^/K^+^-ATPase pump in non-infected and HCMV-infected cells. The α1 (left) and α3 (right) isoforms were detected in cell lysates, nuclear and membrane fractions of non-infected and HCMV-infected cells at 24 hpi. Histone H_3_ and voltage-dependent anion channels (VDACs) were used as controls for the nuclear and membrane fractions, respectively. Supplementary Figure S4: Pattern of α3 staining in non-infected and HCMV-infected HFFs. IFA was performed at 4 h in non-infected (NI) and infected (I) cells. Co-staining used mouse anti-CMV IE1/2 and rabbit anti-α3. Quantification of IE1 and α3 colocalization was determined as described previously. Data represent an average of nine measurements with SD. Significance levels were calculated using an unpaired t-test (*** *p* < 0.001).
