# Supplementary material for: Interspecies Differences in Cytomegalovirus Inhibition by Cardiac Glycosides—A Unique Role of the Alpha3 Isoform of the Na+/K+-ATPase Pump
Source: Viruses. 2025 Mar 11;17(3):398. doi: 10.3390/v17030398 (PMC11946196; doi:10.3390/v17030398)
Supplement: Supplementary file 1 [file viruses-17-00398-s001.zip › viruses-3431893-supplementary.pdf]

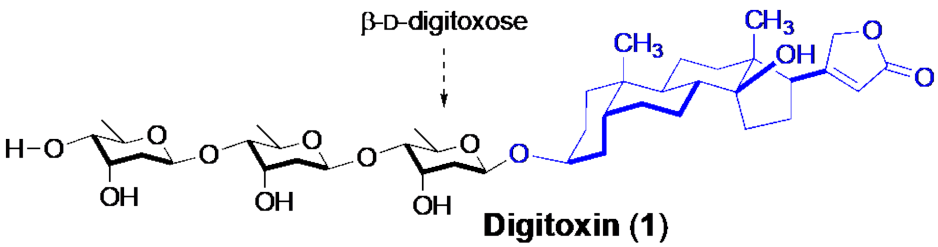

Digitoxin (1)

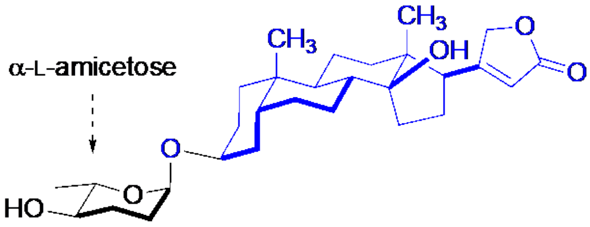

Digitoxigenin α-L-amicetoside (2)

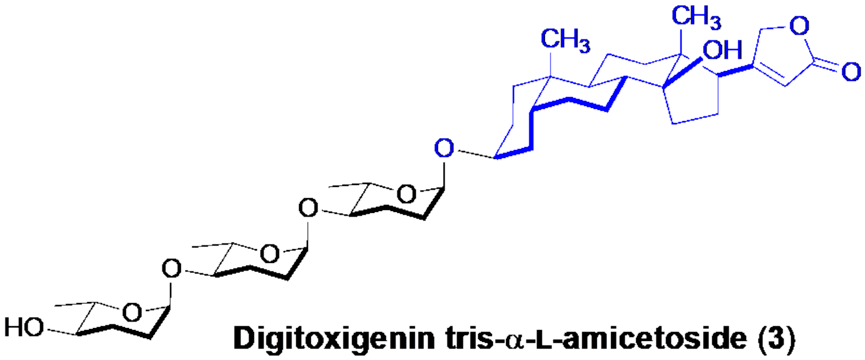

Digitoxigenin tris-α-L-amicetoside (3)

Supplementary Figure 1: Structure of the Cardiac Glycosides. (**1**) Digitoxin, a glycosylated natural product consisting of an aglycon (digitoxigenin in blue) and β-linked 1,4-D-digitoxose trisaccharide. (**2**) Digitoxigenin α-L-amicetoside, a synthetic cardiac glycoside analog consisting of a glycosylated digitoxigenin (blue) with a 3-deoxy-L-sugar (*i.e.*, α-linked L-amicetose). (**3**) Digitoxigenin tris-α-L-amicetoside, a synthetic cardiac glycoside analog consisting of a glycosylated digitoxigenin (blue) with a 3-deoxy-L-sugar trisaccharide (*i.e.*, tris-α-linked L-amicetose).

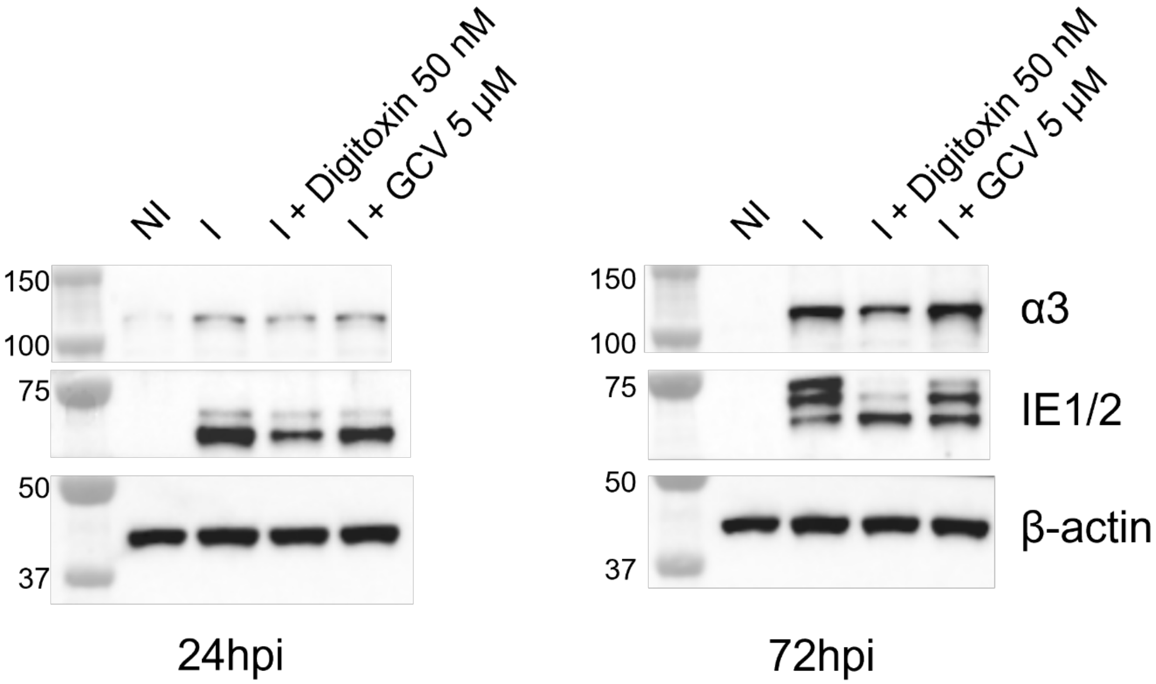

Supplementary Figure 2: α3 expression in TB40E/E infected ARPE-19 cells. The expression of α3 protein was measured in non-infected, HCMV-infected HFFs (TB40E/E, MOI – 3 PFU/cell) and infected treated cells at 24 and 72 hpi. The experiment was performed twice; representative immunoblots are presented. Numbers adjacent to the immunoblots are kDa values.

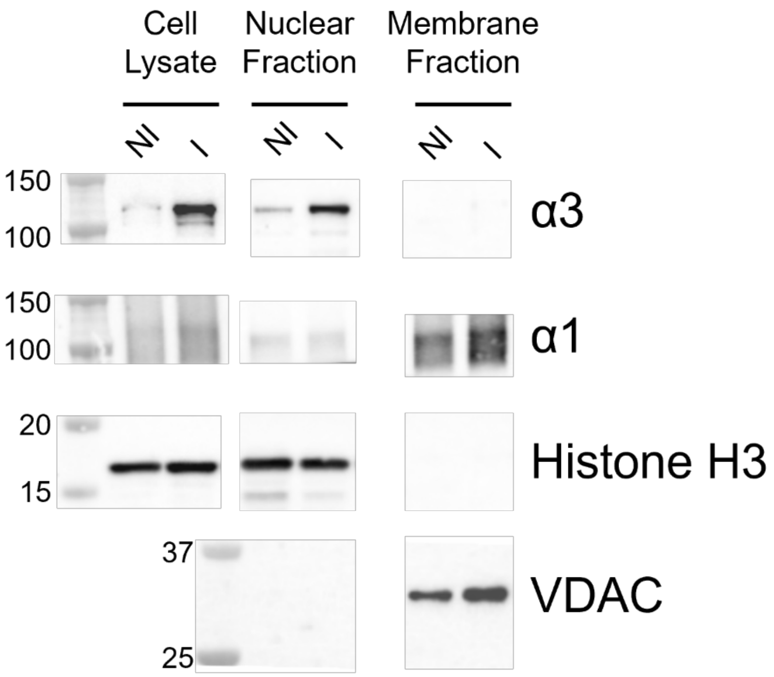

Supplementary Figure 3: localization of the α1 and α3 isoforms of the Na^{+}/K^{+}-ATPase pump in non-infected and HCMV-infected cells. The α1 (left) and α3 (right) isoforms were detected in cell lysates, nuclear and membrane fractions of non-infected and HCMV-infected cells at 24 hpi. Histone H_3 and voltage-dependent anion channel (VDAC) were used as controls for the nuclear and membrane fractions, respectively.

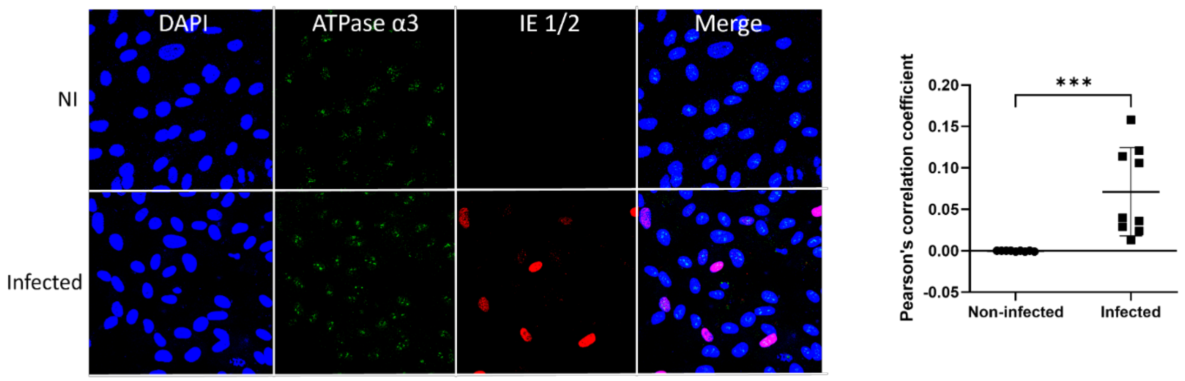

Supplementary Figure 4: Pattern of α3 staining in non-infected and HCMV-infected HFFs. IFA was performed at 4 h in non-infected (NI) and infected (I) cells. Co-staining used mouse anti-CMV IE1/2 and rabbit anti-α3. Quantification of IE1 and α3 colocalization was determined as described previously. Data represent an average of 9 measurements with SD. Significance levels were calculated using an unpaired t-test (***$p < 0.001$).
